# Palm Multidiagnostic of *Mycoplasma pneumoniae*, *Chlamydia pneumoniae*, *Haemophilus influenzae*, and *Streptococcus pneumoniae* Using One-Tube CRISPR/Cas12a

**DOI:** 10.1155/2024/5002521

**Published:** 2024-05-28

**Authors:** Xingchi Kan, Yue Wu, Xiyue Zhang, Zihan Zeng, Chen Lou, Lina Wu, Xingxu Huang, Xinjie Wang, Baicheng Huang

**Affiliations:** ^1^ Zhejiang Lab Hangzhou 311121China; ^2^ Respiratory Department Hangzhou First People's Hospital Hangzhou 310006China; ^3^ School of The First Clinical Medical Sciences Wenzhou Medical University Wenzhou 325035China; ^4^ School of Food Science and Pharmaceutical Engineering Nanjing Normal University Nanjing 210023China; ^5^ Shenzhen Branch Guangdong Laboratory of Lingnan Modern Agriculture Genome Analysis Laboratory of the Ministry of Agriculture and Rural Affairs; Agricultural Genomics Institute at Shenzhen Chinese Academy of Agricultural Sciences Shenzhen 518000China

## Abstract

The recent high incidence of *Mycoplasma pneumoniae* (Mp) infections has raised widespread public health concerns. Therefore, rapid and accurate diagnosis of respiratory pathogenic microbial infections is of paramount importance to provide clinicians with accurate diagnostic insights and guide clinical medication. In response to this urgent need, we developed a one-tube Palm CRISPR/Cas12a Diagnostic (PaCD) method. This method facilitates the rapid detection of Mp infections, as well as three other prevalent respiratory pathogens, *Chlamydia pneumoniae* (Cp), *Haemophilus influenzae* (Hi), and *Streptococcus pneumoniae* (SP). In addition, 3D printing was employed to fabricate a compact detection device that includes a temperature control module set at 39°C and a blue light irradiation module, significantly enhancing the feasibility of point-of-care testing. The PaCD diagnostic process takes only 30 min with a detection limit of 50 copies/test, making it suitable for analysis of sputum and throat swab samples. PaCD demonstrated 100% concordance (72/72) with next-generation sequencing and exhibited high concordance with computed tomography test results. These findings demonstrate the clinical feasibility of PaCD for the rapid and accurate diagnosis of infections caused by four prevalent respiratory pathogens, offering theoretical insights into the versatile application of point-of-care tests for the detection of other respiratory pathogens in various clinical scenarios.

## 1. Introduction

Community-acquired pneumonia (CAP) is a highly contagious disease that poses a serious threat to human health [1]. Respiratory tract infections caused by respiratory pathogens are the main cause of CAP. Among them, *Mycoplasma pneumoniae* (Mp), *Chlamydia pneumoniae* (Cp), *Haemophilus influenzae* (Hi), and *Streptococcus pneumoniae* (SP) are the main pathogens causing CAP [1], with Mp accounting for approximately 40% of CAP infections [2]. Following the COVID-19 pandemic, Mp may pose a significant threat to the respiratory health of the world [3]. Since 2023, there have been many reports of children infected with Mp [4]. Therefore, rapid diagnosis of the pathogens is clinically beneficial for the effective treatment of CAP.

Traditionally, sputum culture has been the “gold standard” for clinical identification of respiratory pathogens, but it is time-consuming [5]. Nowadays, molecular diagnostics are increasing their utility for early point-of-care testing (POCT) [6], like quantitative polymerase chain reaction (qPCR) [7], but it relies on the laboratory environment [8]. The other method, the high-cost next-generation sequencing (NGS) method, provides accurate information of respiratory pathogens, but the samples from bronchoalveolar lavage fluid could cause stress reactions in patients [9]. Therefore, there is a need for more rapid and easy-to-use clinical nucleic acid tests for respiratory bacterial pathogen infections using the easily obtained sputum and throat swabs.

The highly efficient activities of CRISPR Cas13a [10] and Cas12a [11] were reported and widely applied in disease detection [11], characterized by high specificity and sensitivity, rapidity, and low cost. These methods have been validated in the outbreaks of SARS-CoV-2 and human papillomavirus (HPV) [12]. In addition, although the Cas12a-based method for detection of Mp infection has been reported [13], but a rapid and multiple-pathogen detection method for Mp, Cp, Hi, and SP is lacking, which can provide clinicians with an accurate diagnostic basis to control disease progression.

Preamplification is critical in the low copy nucleic acids detection [10]. Compared to PCR, recombinase polymerase amplification (RPA) has the advantages of a constant amplification temperature (37–42°C) and a shorter amplification time, making it suitable for preamplifying nucleic acid molecules in POCT [11]. The feasibility of nucleic acid detection methods combining RPA and CRISPR, including SHERLOCK [10], DETECTR [11], SHINE [14], and CDetection [15], has been developed. The RPA-CRISPR assay is independent of laboratory equipment, providing a user-friendly solution for the rapid pathogen diagnosis in remote areas [16].

Here, we developed a one-tube RPA-CRISPR method to detect the DNA of four pathogens, including Mp, Cp, Hi, and SP, in sputum and throat swab samples. In addition, we utilized 3D printing to fabricate an integrated reaction device, Palm CRISPR Diagnostic (PaCD), equipped with a temperature control module and a blue light detection module, which enabled the assay to achieve sensitive and specific detection of four pathogens in sputum and throat swab within 30 min, with high concordance, sensitivity, and specificity. These findings provide reliable experimental methods for the rapid diagnosis of Mp and help to provide experimental data and technical guidance for the application of POCT in the detection of respiratory pathogens.

## 2. Materials and Methods

### 2.1. Preparation of Clinical Samples

Sputum and throat swab samples were obtained from the Department of Respiratory Medicine, Affiliated Hangzhou First People's Hospital, Zhejiang University School of Medicine. Sputum samples collected in sputum cups were divided into two groups, a negative control group and a positive group, where the positive samples consisted of one or more pathogens (a combination of four pathogens).

Patients were instructed by a healthcare professional before spitting, and 3–5 mL of sputum samples was collected from the patients' deep lungs into cheese-like sputum cups. The sputum was processed according to the instructions of HiPure Sputum DNA Kit (Magen, Guangzhou, China) for DNA extraction.

### 2.2. RPA Primers and CRISPR crRNA Design

We selected the most frequently reported test genes in the genomes of these four pathogens, including the gene sequences of Mp (P1, GenBank accession number: M18639.1) [17], Cp (ompA, GenBank accession number: AF131889.1) [1], Hi (16S-rRNA, GenBank accession number: L42023.1, location of 127,181–127,724), and SP (lytA, GenBank accession number: AJ243399.1) [18] (*Supplementary 1*) from NCBI. The specific method and experimental conditions of PCR were performed according to the previous report [1] (*Supplementary 2*). RPA primer pairs (*Supplementary 3*) (Sangong, Shanghai, China) were designed by EasyDesign (https://crispr.zhejianglab.com/). Three pairs of RPA primers with relatively high scores were selected, and 4–5 crRNA sequences were selected based on the location of the primer in the DNA templates. For the method optimization, the optimal crRNA (*Supplementary 4*) and primer combinations for each pathogen were obtained using online designed RPA primers and crRNA candidates (EasyDesign). In RPA primer screening, the pictures of blue light irradiation were obtained by the LED Transilluminator (Sangon, China), and the fluorescence intensity readouts were obtained by the Spark® multimode microplate reader (Tecan, Switzerland).

### 2.3. Processing of Clinical Sputum Samples

Sputum samples were treated with sputum digest to release DNA (Sputasol method, 100°C for 10 min). The treated samples were used for the one-step RPA-CRISPR assay. The throat swab samples were immersed in nucleic acid rapid lysis solution (GenStar, Beijing, China) and processed at 100°C for 10 min. The processed throat swabs were used for PaCD detection.

### 2.4. The 3D Printing of PaCD

Polylactic acid (PLA) was used as the raw material for 3D printing. The dimensions of the portable inspection device are 10 cm in length, 5 cm in width, and 8 cm in height. The inspection device consists of two functional module areas. At the back of device is a thermostatic heating module, and at the front of it is a fluorescence detection module.

The heating module contains two pads, powered by the USB port, which are made of soft material and can be easily deformed. During operation, the two heating pads sandwich the test tube in the center and maintain the temperature at 39°C to ensure the smooth progress of the nucleic acid amplification reaction.

The fluorescence detection module contains eight blue light sources (luminance 10,000–12,000 mcd; wavelength 460–465 nm), which are connected in parallel and powered by two coin batteries. Each blue light is located directly below the detector tube to ensure full excitation of the fluorescence signal within the detector tube. The device can be placed inside a portable medical bag (16 cm in length, 6 cm in wide, 12 cm in height) for easy transport of the proximity detection device.

### 2.5. One-Tube RPA-CRISPR Reaction Conditions

The Liquid GenDx Basic kits (GenDx, Suzhou, China) were used in the RPA assay, and the reaction system was 30 *μ*L, which was stored in four EP tubes. Tube A contained 10 *μ*L of reaction buffer, 5 *μ*L of amplification enzyme reagent, 1 *μ*L of forward primer (10 *μ*M), 1 *μ*L of reverse primer (10 *μ*M), 2 *μ*L of ssDNA-reporter (Azenta, 12.5 *μ*M), 1.5 *μ*L of 0.05% Tween 20 (Solarbio, Beijing, China), 4.4 *μ*L of DNase/RNase-Free Water (Solarbio, Beijing, China), and 0.1 *μ*L of Recombinant RNase Inhibitor (Novoprotein, Suzhou, China). Tube B contained 1 *μ*L of DNA template. Tube C contained 3 *μ*L of ribonucleoprotein (RNP, including crRNA and Cas12a with the ratio of 1 : 1, 150 nM). Tube D contained 1 *μ*L of MgOC (200 mM). Tubes A, B, C, and D were stored at −80°C. In the detection, the reagent in tube B was added to tube A, and then followed by addition of the reagent from tube D into tube A, and finally, the RNP complex was added to tube A. The reaction conditions were 39°C for 30 min. Note that the RNP complexes in tubes A and C were shaken in a warm bath at 39°C for 30 min before starting the reaction. Finally, the fluorescence signal was measured by PaCD.

Each of the four pairs of RPA forward and reverse primers in the reaction was 1 *μ*L (10 *μ*M), with the ratios of the four pairs of primers being 1 : 1 : 1 : 1 (RPA primer solution was premixed, and 1 *μ*L was aspirated in the detection). For the four plasmids, the total volume was 5 *μ*L, and the ratio was 1 : 1 : 1 : 1 (four equal volumes of plasmid solution were premixed and stored, and 5 *μ*L was aspirated for detection). Plasmids and primers were synthesized by Bioengineering Co. Ltd (Shanghai, China).

### 2.6. Sensitivity and Specificity of RPA-CRISPR

In the limit of detection (LOD) assays, the plasmid templates and GenDx Basic kits were used. The plasmid templates were diluted as 1.0 × 10^6^, 1.0 × 10^5^, 1.0 × 10^4^, 1.0 × 10^3^, 1.0 × 10^2^, 50, and 0 copies/test. After amplification, 5 *μ*L of the RPA product was added to 20 *μ*L of the CRISPR reaction system, and the fluorescence intensity of the reaction was detected after 30 min. In the LOD assay of the sputum sample, negative samples were processed by sputum digestion, and then, templates with different copy numbers (1.0 × 10^6^, 1.0 × 10^5^, 1.0 × 10^4^, 1.0 × 10^3^, 1.0 × 10^2^, 50, and 0 copies/test) were added. Finally, positive sputum DNAs were exposed with the same treatment and used for the detection of the clinical samples. We performed three replicates of the assay to validate the LOD of the PaCD method for the plasmid and sputum samples.

In the specificity assay, all crRNAs of Mp, Cp, Hi, and SP were tested by cross-reaction with the DNA templates of target genes. In addition, the DNA templates extracted from *Klebsiella pneumoniae*, *Pseudomonas aeruginosa*, *Staphylococcus aureus*, *Escherichia faecalis*, *Escherichia coli*, and *Corynebacterium striatum* [18] were tested for the specificity assay using the established quadruple CRISPR method. The pictures of blue light irradiation were obtained by the LED Transilluminator (Sangon, China).

### 2.7. Statistical Analysis

The statistical analysis was performed using Prism 6.0 (GraphPad, USA). Standard deviations and means were calculated using data from at least three identical measurements. Comparisons between multiple datasets were performed using one-way ANOVA. *P*-values < 0.05 were considered statistically significant.

## 3. Results

### 3.1. The Principle of the Palm CRISPR Diagnostic Platform

**Figure 1:**
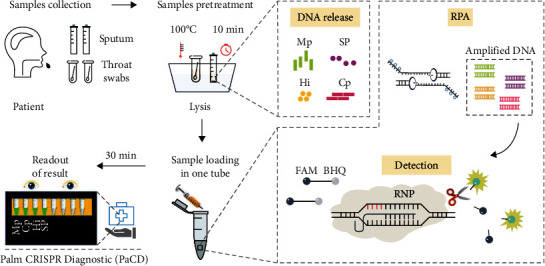
Construction of the RPA-Cas12a-based method for multiplex detection of sputum pathogens. Step 1, sputum samples were collected from patients. Step 2, the Sputasol method treats the sputum samples to release DNA. Step 3, one-tube method detection. Step 4, test results output. The one-tube assay includes RPA and CRISPR reaction components, where RPA can simultaneously amplify four DNA targets, specifically Mp, Cp, Hi, and SP. The Palm assay device, PaCD, integrates reaction heating and result detection, enriching POCT options.

In this study, we developed a compact and operationally efficient RPA-Cas12a-based detection platform (Figure 1), for the detection of the four respiratory pathogens of Mp, Cp, Hi, and SP. This method fused the RPA and the Cas12a-based fluorescent reaction into a single reaction system, reducing the possibility of aerosol contamination and allowing for the simultaneous detection of four respiratory pathogens, increasing the utility of *in situ* detection. In addition, the method used 3D printing technology to provide a portable and rapid detection device, so we named the detection platform as the Palm CRISPR Diagnostic (PaCD).

### 3.2. Optimization of System Parameters for RPA-Cas12a

**Figure 2:**
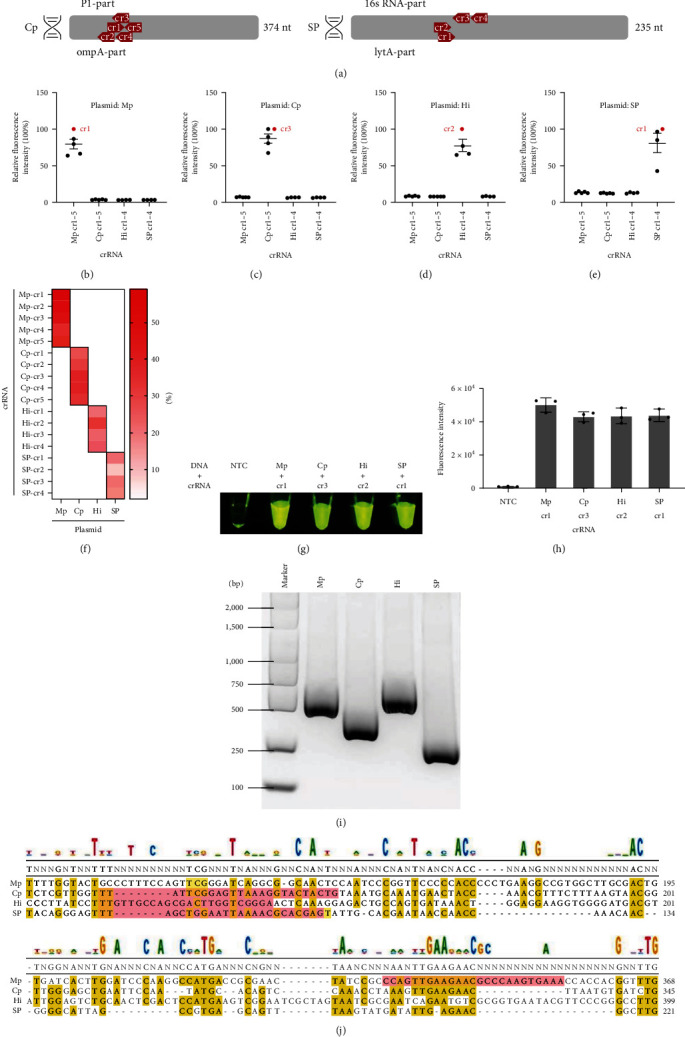
Screening of crRNA. (a) Relative position of crRNAs on target genes. (b–e) Specificity and efficiency screening of crRNAs. (f) Relative values of specificity and efficiency in heat map analysis of crRNAs. (g, h) One-tube fluorescence detection and statistics of optimal crRNA. (i) Nucleic acid gel validation of target genes. Marker, DL2000. (j) Sequence of Mp, Cp, Hi, and SP optimal crRNAs. NTC, no template control.

To optimize the RPA-Cas12a method, the concentration of ssDNA-FQ reporter in the Cas12a reaction was set at 25 *μ*M according to the previous report [19], and the results showed a significant fluorescence signal intensity. Next, in the crRNA selection, 4–5 crRNAs were designed for the four pathogens (Figure 2(a)), and then, the crRNA with the strongest cleavage activity (fluorescence intensity) was selected, Mp-crRNA1, Cp-crRNA3, Hi-crRNA2, and SP-crRNA1 (Figure 2(g), 2(h), 2(i), and 2(j)).

**Figure 3:**
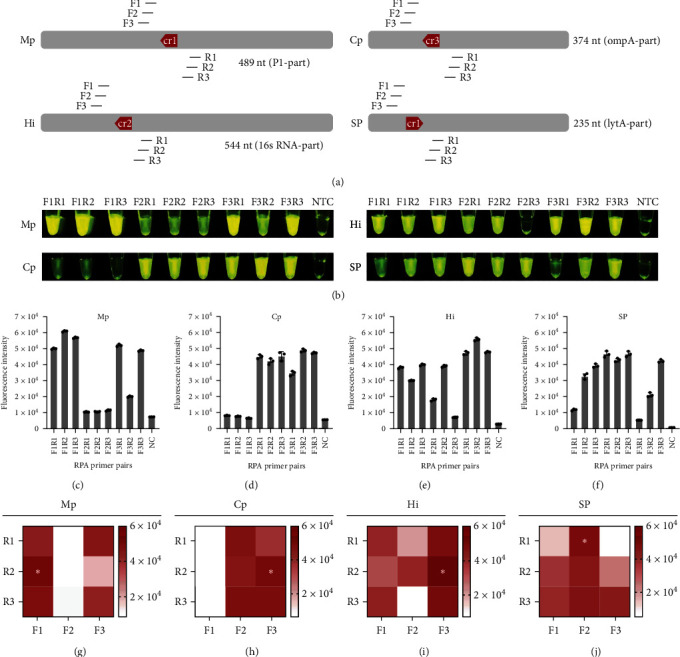
Screening of the RPA primers. (a) Relative position of RPA primers and screening of optimal primers. (b) One-tube reactions corresponding to different RPA primer combinations. (c–f) Statistics of fluorescence intensity of different RPA primer combinations corresponding to a one-tube reaction. (g–j) Heat map method for screening the optimal primer combinations. NTC, no template control.

For RPA primer selection, three pairs of RPA primers were designed for each target gene in this study (Figure 3(a)). A 3 × 3 matrix was used to select the RPA primer pair with the strongest fluorescence signal (Figure 3(b)). The results indicated that the optimal combinations of RPA primer pairs were Mp-F1-R2 (Figures 3(c) and 3(g)), Cp-F3-R2 (Figures 3(d) and 3(h)), Hi-F3-R2 (Figures 3(e) and 3(i)), and SP-F2-R1 (Figures 3(f) and 3(j)), respectively.

### 3.3. Sensitivity and Specificity of PaCD

**Figure 4:**
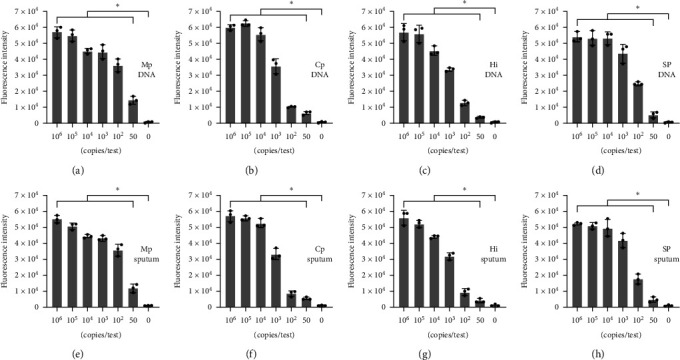
Sensitivity analysis of Cas12a-based fluorescent method of four respiratory pathogens. (a–d) Detection sensitivity of Mp, Cp, Hi, and SP in plasmid samples, respectively. (e–h) Detection sensitivity of Mp, Cp, Hi, and SP in sputum samples, respectively. Three replicates of the assay were performed to validate the LOD of the PaCD method for the plasmid and sputum samples.  ^*∗*^ represent statistically significant differences between groups (*p* < 0.05).

To evaluate the sensitivity of this method, LOD tests were performed for each target gene. The results showed that the LOD for the detection of Mp, Cp, Hi, and SP by PaCD all reached 50 copies/test (Figure 4(a), 4(b), 4(c), and 4(d)).

To eliminate the interference of sputum on the detection sensitivity, further determination of the LOD in sputum was performed. The Sputasol-treated negative sputum samples that were mixed with the different concentration of plasmids of Mp, Cp, Hi, and SP were detected. The results showed that the LOD of Mp, Cp, Hi, and SP after sputum treatment all reached 50 copies/test, respectively (Figure 4(e), 4(f), 4(g), and 4(h)), which was consistent with the results of LOD using plasmids as the templates.

In the PaCD specificity assay, all crRNAs of Mp, Cp, Hi, and SP reacted only with the corresponding targets, and no cross-reactions were observed (Figure 2(b), 2(c), 2(d), 2(e), and 2(f)). For other bacteria, only the positive controls yielded positive results, whereas negative results were obtained for *Klebsiella pneumoniae*, *Pseudomonas aeruginosa*, *Staphylococcus aureus*, *Escherichia faecalis*, *Escherichia coli*, *Corynebacterium striatum*, and the no template control (*Supplementary 2*). Therefore, these results indicate that the PaCD assay is highly specific for the detection of Mp, Cp, Hi, and SP.

### 3.4. 3D Printing of PaCD and Its Application in Patient Samples

**Figure 5:**
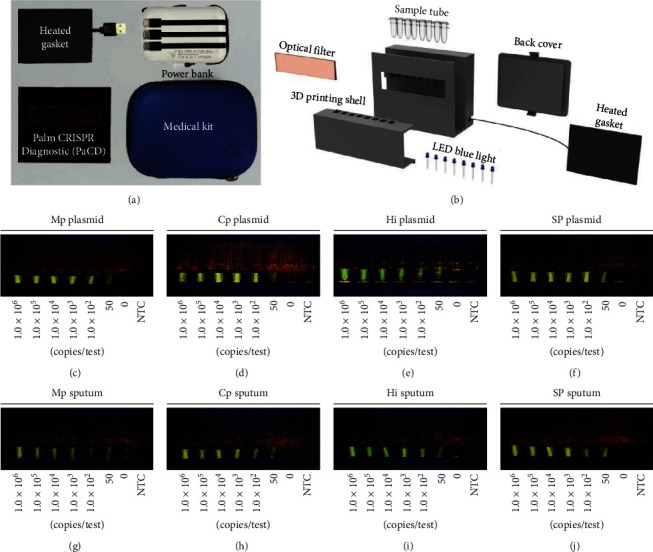
PaCD-based point-of-care detection. (a) Main components of PaCD. (b) The inner structure of PaCD. (c–f) Sensitivity of PaCD for detection of plasmid samples performed with multiple dilution (copies/test) in blue light (460–465 nm). (g–j) Sensitivity of PaCD for sputum samples performed with multiple dilution (copies/test) in blue light (460–465 nm). NTC, no template control.

To expand the application scenarios of the RPA-Cas12a method and improve its POCT applicability, 3D printing technology was used to fabricate a micro RPA-Cas12a detection workstation equipped with a microheating module and ultraviolet irradiation system (Figures 5(a) and 5(b)), PaCD. When tested at concentrations ranging from 0 to 1.0 × 10^6^ copies/test, 50 copies/test of Mp, Cp, Hi, and SP target genes in plasmid and sputum samples could be detected by PaCD (Figure 5(c), 5(d), 5(e), 5(f), 5(g), 5(h), 5(i), and 5(j)).

### 3.5. Clinical Validation of PaCD

**Figure 6:**
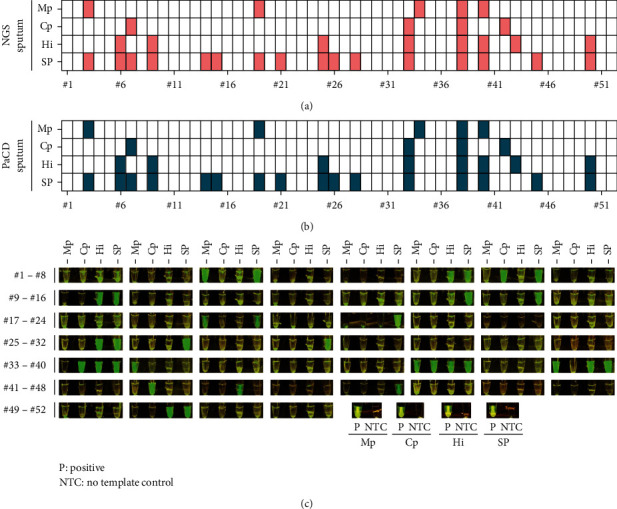
Sputum clinical sample validation of PaCD. (a) NGS detection results of 52 clinical sputum samples. (b) Results of PaCD detection in 52 clinical sputum samples. (c) Results of PaCD detection in 52 clinical sputum samples in blue light. P, positive control; NTC, no template control.

Sputum sample collection in clinical practice has the advantage of being noninvasive sampling and convenient sampling for patients. Therefore, PaCD focuses on the detection of Mp, Cp, Hi, and SP in sputum samples. This experiment was double-blinded. Clinical samples with known positive and negative results were randomly mixed and numbered. A total of 52 clinical sputum samples with NGS test reports (Figure 6) were tested, of which 33 were negative and 19 were positive. The positive samples contained single or multiple infections with Mp, Cp, Hi, and SP. The results of the PaCD in 52 clinical sputum samples showed that 19 positive samples were detected (Figure 6(b)), with a sensitivity of 100% (19/19) based on the NGS result, and 33 negative samples were detected, with a specificity of 100% (33/33) compared with the NGS result. The overall accuracy of the test was 100% (52/52).

**Figure 7:**
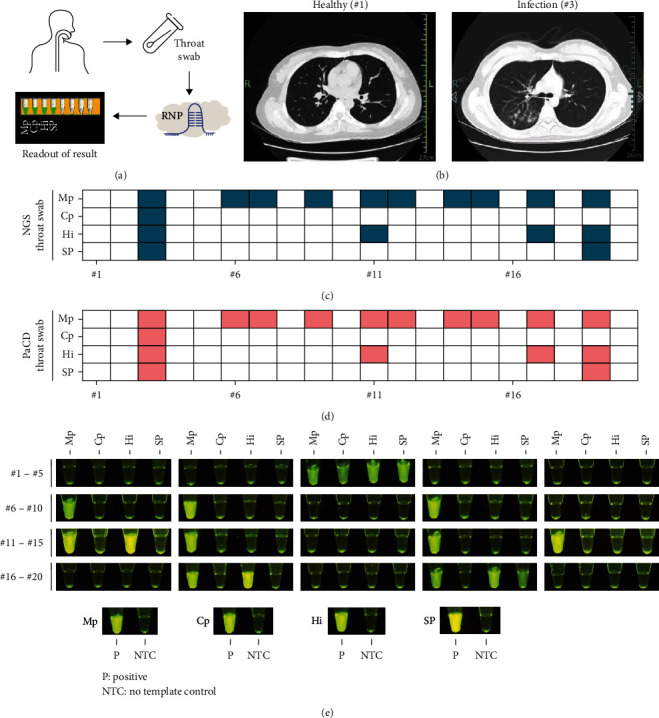
Throat swab clinical sample validation of PaCD. (a) Schematic diagram of clinical samples for PaCD detection of throat swab samples. (b) CT test results of the sample #1 (healthy) and #3 (infected). (c) NGS detection results of 20 clinical throat swab samples. (d) Results of PaCD detection in 20 clinical throat swab samples. (e) Results of PaCD detection in 20 clinical throat swab samples in blue light. NTC, no template control. The reaction components of the NTC group were consistent with the other template-containing reaction groups, except that the template used H_2_O instead.

To address the threat posed by Mp, it is important to increase the application scenarios for Mp detection. Therefore, this study investigated the detection effect of PaCD on throat swabs. Because the sputum collection requires the effective patient cooperation, children often cannot effectively cooperate with the sputum collection, but throat swabs can effectively collect samples of respiratory pathogenic microbial infections in children.  Figure 7(a) shows a schematic diagram of the PaCD detection process for pathogens such as Mp in throat swab samples.

A total of 20 throat swab samples with NGS results were tested, of which 10 were negative and 10 were positive (Figure 7(c)). The results of the patient's CT diagnosis (Figure 7(b) and *Supplementary 2*) were used to preliminarily determine whether the patient has pneumonia. The results of the PaCD showed that 10 positive samples were detected, with a sensitivity of 100% (10/10) based on the NGS result, and 10 negative samples were detected, with a specificity of 100% (10/10) compared with the NGS result (Figures 7(d) and 7(e)) (*Supplementary 5*).

This method allows the successful detection of Mp, Cp, Hi, and SP in a one-tube system for sputum and throat swab samples, providing an increased advantage over the current one-step detection system. In addition, PaCD provides valuable experimental data and technical guidance for the accurate and rapid diagnosis of Mp infection.

## 4. Discussion

On-site, rapid, and accurate nucleic acid testing is clinically important as it provides an excellent guide to the prognostic assessment of the disease [20], as confirmed by the high infection of Mp [4, 21]. Rapid and accurate diagnosis of Mp, Cp, Hi, and SP is the first step to getting effective treatment. Therefore, in this study, a one-tube detection method based on RPA and Cas12a was developed for the detection of four pathogens simultaneously. The four target genes of the pathogens with relatively high infection risk and more severe effects were selected for detection: P1 (for Mp), ompA (for Cp), 16S-rRNA (for Hi), and lytA (for SP).

NGS is faster and more accurate for nucleic acid testing [22], while the need for specialized operators and instrumentation limits its application. Compared to NGS, the constant reaction temperature of RPA (37-42°C) is independent of specialized instrumentation [23], making the experimental procedure simple, time-saving, and efficient, and allowing it to be easily linked to other assays [23, 24]. Therefore, here, the time-efficient, one-tube, quadruple detection method based on RPA and Cas12a for the detection of sputum pathogens is low-cost, noninvasive, and minimally stressful for patients. The results of the optimized RPA-Cas12a detection system are consistent with those of the NGS assay, but it has significant operational simplicity and is suitable for rapid clinical identification, could effectively detect the above pathogens in sputum with high sensitivity (50 copies/test).

A rapid, accurate, portable, multiplexed test with high sensitivity comparable to PCR is needed to broaden the scope of disease surveillance and popularize it in remote areas with poor medical conditions. The RPA-CRISPR reaction system has a complex composition of reaction reagents, including polyethylene glycol, which is an important factor causing the crowding effect of the macromolecules. Polyethylene glycol leads to poor mobility of the macromolecules and tends to cause the rapid depletion of local reagents, inhibiting the subsequent cascade amplification reaction, which is one of the reasons for the poor reproducibility of the CRISPR detection system [25]. To address this issue, we attempted to add a liquid surfactant to promote flow between the macromolecules and further reduce the reaction viscosity, and we found that the stability of the one-step detection system was improved after the addition of 0.05% Tween 20.

The 3D printing-assisted detection methods adapted with RPA-Cas12a have been reported, facilitating the application of POCT in multiple scenarios [26]. Inspired by this, we used 3D printing technology to customize an integrated device for the rapid detection method of RPA-Cas12a, which was equipped with heating modules at 39°C (for RPA reaction) and 37.5°C (for nonspecific side cleavage of CRISPR) and a blue light system used to stimulate the ssDNA-reporter to generate fluorescent signals. Our results showed that the integrated device was able to effectively detect four pathogens in sputum, and the time of the whole reaction was controlled within 30 min. This device greatly enriches the POCT application scenarios, as the blue light and thermostatic heating system are difficult to provide in remote rural areas, which is solved by this integrated device. In addition, due to its small size, the device can also be placed in the home medical insurance, which is also conducive to long-distance transportation, which could help alleviate the lack of detection resources in remote areas. As a visual inspection method for sample detection, consideration could be given to objectifying and automating the determination standard of the method if portable equipment for objective measurement is subsequently developed.

When testing clinical samples, the PaCD method showed a concordance rate of 100% (72/72). The sensitivity of PaCD (50 copies/test) still has potential for improvement compared to other reports [18, 26]. Compared with the current one-step detection system, PaCD can detect four target genes in the one-step detection system, effectively distinguishing four pathogens, and the sensitivity is relatively close to the LOD of the current one-step method [18]. In addition, PaCD has a higher multiplexing potential of carrying capacity, which means that up to 8 targets can be realized, leaving enough space for multiplex development. A report showed that its rapid test has a maximum number of six tests, and PaCD has two more target gene detection capabilities compared to its rapid test method [26], showing that the fluorescence detection of PaCD has good detection capability of Mp, Cp, Hi, and SP.

The next step was the optimization of the detection equipment to enable better fluorescence signal acquisition than is currently possible, avoiding interference from background fluorescence signals, such as the use of mobile phone photography to automatically identify fluorescence signal intensity characteristics.

## 5. Conclusion

In summary, we developed a one-tube Palm CRISPR/Cas12a Diagnostic (PaCD) method for the rapid detection of respiratory pathogens (Mp, Cp, Hi, and SP), and this method is clinically time-saving, rapid, and accurate. 3D printing technology enhances the point-of-care utility of this method by constructing a temperature control module and a blue light irradiation module. The PaCD diagnostic process takes only 30 min with a detection limit of 50 copies/test, making it suitable for analyzing sputum and throat swab samples. PaCD covers the pathogens with a relatively high risk of infection, providing experimental data and technical guidance for rapid and accurate diagnosis of respiratory pathogen infections.

## Data Availability

The data supporting the findings of this study can be obtained on reasonable request.

## Supplementary Materials

Supplementary 1 Table S1: the nucleotide sequence of target. Table S2: the sequence list of PCR primers for target gene amplification. Table S3: the sequence of RPA primers. Table S4: the sequence of crRNAs. Table S5: comparison of NGS and PaCD (with fluorescence test) results of clinical samples.

Supplementary 2 Figure S1: specific assay of the one-step RPA-CRISPR method in different bacterial strains. The one-step reaction system contains four preferred crRNAs that initiate the reaction when the DNA template binds to the crRNA. P, positive (add the DNA template corresponding to crRNA); NTC, no template control. 1, *Klebsiella pneumoniae*; 2, *Pseudomonas aeruginosa*; 3, *Staphylococcus aureus*; 4, *Escherichia faecalis*; 5, *Escherichia coli*; 6 *Corynebacterium striatum*. Figure S2: results of CT testing of throat swab samples. A total of 18 of the 20 throat swab samples are shown, and the remaining 2 samples representing healthy (#1) and infected (#3) are shown in Figure 7(b).
