# Supplementary material for: Palm Multidiagnostic of Mycoplasma pneumoniae, Chlamydia pneumoniae, Haemophilus influenzae, and Streptococcus pneumoniae Using One-Tube CRISPR/Cas12a
Source: Transbound Emerg Dis. 2024 May 28;2024:5002521. doi: 10.1155/2024/5002521 (PMC12019929; doi:10.1155/2024/5002521)
Supplement: Supplementary 1 — Table S1: the nucleotide sequence of target. Table S2: the sequence list of PCR primers for target gene amplification. Table S3: the sequence of RPA primers. Table S4: the sequence of crRNAs. Table S5: comparison of NGS and PaCD (with fluorescence test) results of clinical samples. [file 5002521.f1.docx]

**Supplementary Table 1. The nucleotide sequence of target**

| **Target gene** | **Sequence（5’-3’）** |
| --- | --- |
| *Mycoplasma pneumoniae*  (Mp)  P1  M18639.1  489 bp | CGGACTCGGAGGACAATGGTCAGCTGCAGTTAGAAAAAAATGATCTCGCCAACGCTCCCATTAAGCGGAGCGAGGAGTCGGGTCAGTCCGTCCAACTCAAGGCGGACGATTTTGGTACTGCCCTTTCCAGTTCGGGATCAGGCGGCAACTCCAATCCCGGTTCCCCCACCCCCTGAAGGCCGTGGCTTGCGACTGAGCAAATTCACAAGGACCTCCCCAAATGATCCGCCTCGATCCTGATTCTGTACGATGCGCCTTATGCGCGCAACCGTACCGCCATTGACCGCGTTGATCACTTGGATCCCAAGGCCATGACCGCGAACTATCCGCCCAGTTGAAGAACGCCCAAGTGAAACCACCACGGTTTGTGGGACTGAAAGGCGCGCGATGTTTTGCTCCAAACCACCGGGTTCTTCAACCCGCGCCGCCACCCCGAGTGGTTTGATGGCGGGCAGACGGTCGCGGATAACGAAAAGACCGGGTTTGATG |
| *Chlamydia pneumoniae*  (Cp)  ompA  AF131889.1  374 bp | TTTACACGATGCAGAGTGGTTCACTAATGCAGGCTTCATTGCCTTAAACATTTGGGATCGCTTTGATGTTTTCTGTACTTTAGGAGCTTCTAATGGTTACATTAGAGGAAACTCTACAGCGTTCAATCTCGTTGGTTTATTCGGAGTTAAAGGTACTACTGTAAATGCAAATGAACTACCAAACGTTTCTTTAAGTAACGGAGTTGTTGAACTTTACACAGACACCTCTTTCTCTTGGAGCGTAGGCGCTCGTGGAGCCTTATGGGAATGCGGTTGTGCAACTTTGGGAGCTGAATTCCAATATGCACAGTCCAAACCTAAAGTTGAAGAACTTAATGTGATCTGTAACGTATCGCAATTCTCTGTAAACAAAC |
| *Haemophilus influenzae*  (Hi)  16S-rRNA  L42023.1  (127,181-127,724)  544 bp | ACATCCTAAGAAGAGCTCAGAGATGAGCTTGTGCCTTCGGGAACTTAGAGACAGGTGCTGCATGGCTGTCGTCAGCTCGTGTTGTGAAATGTTGGGTTAAGTCCCGCAACGAGCGCAACCCTTATCCTTTGTTGCCAGCGACTTGGTCGGGAACTCAAAGGAGACTGCCAGTGATAAACTGGAGGAAGGTGGGGATGACGTCAAGTCATCATGGCCCTTACGAGTAGGGCTACACACGTGCTACAATGGCGTATACAGAGGGAAGCGAAGCTGCGAGGTGGAGCGAATCTCATAAAGTACGTCTAAGTCCGGATTGGAGTCTGCAACTCGACTCCATGAAGTCGGAATCGCTAGTAATCGCGAATCAGAATGTCGCGGTGAATACGTTCCCGGGCCTTGTACACACCGCCCGTCACACCATGGGAGTGGGTTGTACCAGAAGTAGATAGCTTAACCTTTTGGAGGGCGTTTACCACGGTATGATTCATGACTGGGGTGAAGTCGTAACAAGGTAACCGTAGGGGAACCTGCGGTTGGATCACCT |
| *Streptococcus* *pneumoniae*  (SP)  lytA  AJ243399.1  235 bp | TGACGGACTACCGCCTTTATATCGAACTCTTACGCAATCTAGCAGATGAAGCAGGTTTGCCGAAAACGCTTGATACAGGGAGTTTAGCTGGAATTAAAACGCACGAGTATTGCACGAATAACCAACCAAACAACCACTCAGACCATGTGGATCCATACCCTTACTTGGCAAAATGGGGCATTAGCCGTGAGCAGTTTAAGTATGATATTGAGAACGGCTTGACGATTGAAACAGG |

**Supplementary Table 2. The sequence list of PCR primers for target gene amplification**

| **Primer name** | **Sequence** |
| --- | --- |
| Mp-P1-F1 | ACTCGGAGGACAATGGTCAG |
| Mp-P1-R1 | CAAACCCGGTCTTTTCGTTA |
| Cp-ompA-F1 | ACACGATGCAGAGTGGTTCA |
| Cp-ompA-R1 | TGTTTACAGAGAATTGCGATACG |
| Hi-16S-rRNA-F1 | TCCTAAGAAGAGCTCAGAGAT |
| Hi-16S-rRNA-R1 | TGATCCAACCGCAGGTTCC |
| SP-lytA-F1 | CGGACTACCGCCTTTATATCG |
| SP-lytA-R1 | GTTTCAATCGTCAAGCCGTT |

**Supplementary Table 3. The sequence of RPA primers**

| **Primer** | **Sequence (5’-3’)** | **Primer** | **Sequence (5’-3’)** |
| --- | --- | --- | --- |
| **Mp-F1** | **TATCCGCCCAGTTGAAGAACGCCCAAGTGA** | **Mp-R1** | TTTCGTTATCCGCGACCGTCTGCCCGCCAT |
| **Mp-F2** | AAGAACGCCCAAGTGAAACCACCACGGTTT | **Mp-R2** | **GTTATCCGCGACCGTCTGCCCGCCATCAAA** |
| **Mp-F3-O** | TTGAAGAACGCCCAAGTGAAACCACCACGG | **Mp-R3** | ATCCGCGACCGTCTGCCCGCCATCAAACCA |
| **Cp-F1** | ACTTTAGGAGCTTCTAATGGTTACATTAGA | **Cp-R1** | TAAGGCTCCACGAGCGCCTACGCTCCAAGA |
| **Cp -F2** | TGTACTTTAGGAGCTTCTAATGGTTACATT | **Cp-R2** | **AATTCAGCTCCCAAAGTTGCACAACCGCAT** |
| **Cp -F3** | **CTTTAGGAGCTTCTAATGGTTACATTAGAG** | **Cp-R3** | GTTGCACAACCGCATTCCCATAAGGCTCCA |
| **Hi-F1** | **GGTGCTGCATGGCTGTCGTCAGCTCGTGTT** | **Hi-R1** | GCCATGATGACTTGACGTCATCCCCACCTT |
| **Hi-F2** | TTAGAGACAGGTGCTGCATGGCTGTCGTCA | **Hi-R2** | **ATTGTAGCACGTGTGTAGCCCTACTCGTAA** |
| **Hi-F3-O** | CTTCGGGAACTTAGAGACAGGTGCTGCATG | **Hi-R3** | TGACTTGACGTCATCCCCACCTTCCTCCAG |
| SP**-F1** | ATGAAGCAGGTTTGCCGAAAACGCTTGATA | **SP-R1** | **AATATCATACTTAAACTGCTCACGGCTAAT** |
| SP**-F2** | **AGATGAAGCAGGTTTGCCGAAAACGCTTGA** | **SP-R2** | AATCGTCAAGCCGTTCTCAATATCATACTT |
| **SP-F3** | ACTCTTACGCAATCTAGCAGATGAAGCAGG | **SP-R3** | TACTTAAACTGCTCACGGCTAATGCCCCAT |

Note: The sequences of optimal RPA primer pairs for the targets are shown in bold.

**Supplementary Table 4. The sequence of crRNAs**

| **crRNA** | **Sequence** |
| --- | --- |
| Mp-cr1 | UAAUUUCUACUAAGUGUAGAUACUUGGGCGUUCUUCAACUGG |
| Mp-cr2 | UAAUUUCUACUAAGUGUAGAUUGGGACUGAAAGGCGCGCGAU |
| Mp-cr3 | UAAUUUCUACUAAGUGUAGAUAGUCCCACAAACCGUGGUGGU |
| Mp-cr4 | UAAUUUCUACUAAGUGUAGAUCUCCAAACCACCGGGUUCUUC |
| Mp-cr5 | UAAUUUCUACUAAGUGUAGAUGAGCAAAACAUCGCGCGCCUU |
| Cp-cr1 | UAAUUUCUACUAAGUGUAGAUUUCGGAGUUAAAGGUACUACU |
| Cp-cr2 | UAAUUUCUACUAAGUGUAGAUACUCCGAAUAAACCAACGAGA |
| Cp-cr3 | UAAUUUCUACUAAGUGUAGAUCAGUAGUACCUUUAACUCCGA |
| Cp-cr4 | UAAUUUCUACUAAGUGUAGAUCAUUUACAGUAGUACCUUUAA |
| Cp-cr5 | UAAUUUCUACUAAGUGUAGAUGUAGUUCAUUUGCAUUUACAG |
| Hi-cr1 | UAAUUUCUACUAAGUGUAGAUACAACACGAGCUGACGACAGC |
| Hi-cr2 | UAAUUUCUACUAAGUGUAGAUUUGCCAGCGACUUGGUCGGGA |
| Hi-cr3 | UAAUUUCUACUAAGUGUAGAUAGUUCCCGACCAAGUCGCUGG |
| Hi-cr4 | UAAUUUCUACUAAGUGUAGAUUCACUGGCAGUCUCCUUUGAG |
| SP-cr1 | UAAUUUCUACUAAGUGUAGAUGCUGGAAUUAAAACGCACGAG |
| SP-cr2 | UAAUUUCUACUAAGUGUAGAUAUUCCAGCUAAACUCCCUGUA |
| SP-cr3 | UAAUUUCUACUAAGUGUAGAUGUUGGUUAUUCGUGCAAUACU |
| SP-cr4 | UAAUUUCUACUAAGUGUAGAUCCAAGUAAGGGUAUGGAUCCA |

**Supplementary Table 5. Comparison of NGS and PaCD (with fluorescence test) results of clinical samples**

| Sputum  No. | NGS | | | |  | PaCD | | | |  | Fluorescence intensity | | | |
| --- | --- | --- | --- | --- | --- | --- | --- | --- | --- | --- | --- | --- | --- | --- |
|  | Mp | Cp | Hi | SP |  | Mp | Cp | Hi | SP |  | Mp | Cp | Hi | SP |
| 1 | - | - | - | - |  | - | - | - | - |  | 1185 | 1134 | 2593 | 2362 |
| 2 | - | - | - | - |  | - | - | - | - |  | 2827 | 1597 | 1009 | 1788 |
| 3 | + | - | - | + |  | + | - | - | + |  | 57330 | 1646 | 1748 | 56770 |
| 4 | - | - | - | - |  | - | - | - | - |  | 1843 | 1988 | 1776 | 2890 |
| 5 | - | - | - | - |  | - | - | - | - |  | 1738 | 1803 | 1842 | 1795 |
| 6 | - | - | + | + |  | - | - | + | + |  | 1829 | 1701 | 45811 | 48260 |
| 7 | - | + | - | + |  | - | + | - | + |  | 1895 | 59760 | 1880 | 52020 |
| 8 | - | - | - | - |  | - | - | - | - |  | 1645 | 1739 | 1823 | 1754 |
| 9 | - | - | + | + |  | - | - | + | + |  | 1752 | 1757 | 52305 | 51407 |
| 10 | - | - | - | - |  | - | - | - | - |  | 3144 | 1821 | 1848 | 2039 |
| 11 | - | - | - | - |  | - | - | - | - |  | 1796 | 1809 | 1760 | 1692 |
| 12 | - | - | - | - |  | - | - | - | - |  | 1676 | 1644 | 1745 | 1711 |
| 13 | - | - | - | - |  | - | - | - | - |  | 1764 | 1876 | 1820 | 1893 |
| 14 | - | - | - | + |  | - | - | - | + |  | 2376 | 2489 | 2845 | 60588 |
| 15 | - | - | - | + |  | - | - | - | + |  | 1680 | 1767 | 1859 | 48890 |
| 16 | - | - | - | - |  | - | - | - | - |  | 3868 | 1964 | 2108 | 2753 |
| 17 | - | - | - | - |  | - | - | - | - |  | 1883 | 1867 | 1754 | 1831 |
| 18 | - | - | - | - |  | - | - | - | - |  | 1766 | 3718 | 1884 | 1769 |
| 19 | + | - | - | + |  | + | - | - | + |  | 39630 | 1947 | 4271 | 32147 |
| 20 | - | - | - | - |  | - | - | - | - |  | 1729 | 1728 | 1775 | 1866 |
| 21 | - | - | - | + |  | - | - | - | + |  | 1781 | 1705 | 1827 | 53429 |
| 22 | - | - | - | - |  | - | - | - | - |  | 1792 | 1924 | 1914 | 1978 |
| 23 | - | - | - | - |  | - | - | - | - |  | 1678 | 1740 | 1770 | 1709 |
| 24 | - | - | - | - |  | - | - | - | - |  | 1665 | 1654 | 1673 | 1712 |
| 25 | - | - | + | + |  | - | - | + | + |  | 1185 | 1134 | 55931 | 53624 |
| 26 | - | - | - | + |  | - | - | - | + |  | 1788 | 1597 | 1009 | 52827 |
| 27 | - | - | - | - |  | - | - | - | - |  | 1733 | 1777 | 1748 | 1646 |
| 28 | - | - | - | + |  | - | - | - | + |  | 1843 | 1988 | 1776 | 45481 |
| 29 | - | - | - | - |  | - | - | - | - |  | 1738 | 1803 | 1842 | 1795 |
| 30 | - | - | - | - |  | - | - | - | - |  | 1829 | 1701 | 1811 | 1826 |
| 31 | - | - | - | - |  | - | - | - | - |  | 1895 | 1976 | 1880 | 2020 |
| 32 | - | - | - | - |  | - | - | - | - |  | 1645 | 1739 | 1823 | 1754 |
| 33 | - | + | + | + |  | - | + | + | + |  | 1752 | 50757 | 42305 | 41407 |
| 34 | + | - | - | - |  | + | - | - | - |  | 33144 | 1821 | 1848 | 2039 |
| 35 | - | - | - | - |  | - | - | - | - |  | 1796 | 1809 | 1760 | 1692 |
| 36 | - | - | - | - |  | - | - | - | - |  | 1676 | 1644 | 1745 | 1711 |
| 37 | - | - | - | - |  | - | - | - | - |  | 1764 | 1876 | 1820 | 1893 |
| 38 | + | + | + | + |  | + | + | + | + |  | 42376 | 49891 | 48453 | 47588 |
| 39 | - | - | - | - |  | - | - | - | - |  | 1680 | 1767 | 1859 | 1889 |
| 40 | + | - | + | + |  | + | - | + | + |  | 58682 | 1964 | 51080 | 57534 |
| 41 | - | - | - | - |  | - | - | - | - |  | 1883 | 1867 | 1754 | 1831 |
| 42 | - | + | - | - |  | - | + | - | - |  | 1766 | 47183 | 1884 | 1769 |
| 43 | - | - | + | - |  | - | - | + | - |  | 1947 | 1963 | 32713 | 2147 |
| 44 | - | - | - | - |  | - | - | - | - |  | 1729 | 1728 | 1775 | 1866 |
| 45 | - | - | - | + |  | - | - | - | + |  | 1704 | 1659 | 1722 | 31866 |
| 46 | - | - | - | - |  | - | - | - | - |  | 1792 | 1924 | 1914 | 1978 |
| 47 | - | - | - | - |  | - | - | - | - |  | 1678 | 1740 | 1770 | 1709 |
| 48 | - | - | - | - |  | - | - | - | - |  | 1665 | 1654 | 1673 | 1712 |
| 49 | - | - | - | - |  | - | - | - | - |  | 1165 | 3294 | 1796 | 1813 |
| 50 | - | - | + | + |  | - | - | + | + |  | 1147 | 1169 | 47695 | 43527 |
| 51 | - | - | - | - |  | - | - | - | - |  | 338 | 551 | 428 | 420 |
| 52 | - | - | - | - |  | - | - | - | - |  | 1156 | 1013 | 3244 | 1856 |

Note: “+”, positive; “-”, negative.
