# Supplementary material for: Palm Multidiagnostic of Mycoplasma pneumoniae, Chlamydia pneumoniae, Haemophilus influenzae, and Streptococcus pneumoniae Using One-Tube CRISPR/Cas12a
Source: Transbound Emerg Dis. 2024 May 28;2024:5002521. doi: 10.1155/2024/5002521 (PMC12019929; doi:10.1155/2024/5002521)
Supplement: Supplementary 2 — Figure S1: specific assay of the one-step RPA-CRISPR method in different bacterial strains. The one-step reaction system contains four preferred crRNAs that initiate the reaction when the DNA template binds to the crRNA. P, positive (add the DNA template corresponding to crRNA); NTC, no template control. 1, Klebsiella pneumoniae; 2, Pseudomonas aeruginosa; 3, Staphylococcus aureus; 4, Escherichia faecalis; 5, Escherichia coli; 6 Corynebacterium striatum. Figure S2: results of CT testing of throat swab samples. A total of 18 of the 20 throat swab samples are shown, and the remaining 2 samples representing healthy (#1) and infected (#3) are shown in Figure 7(b). [file 5002521.f2.docx]

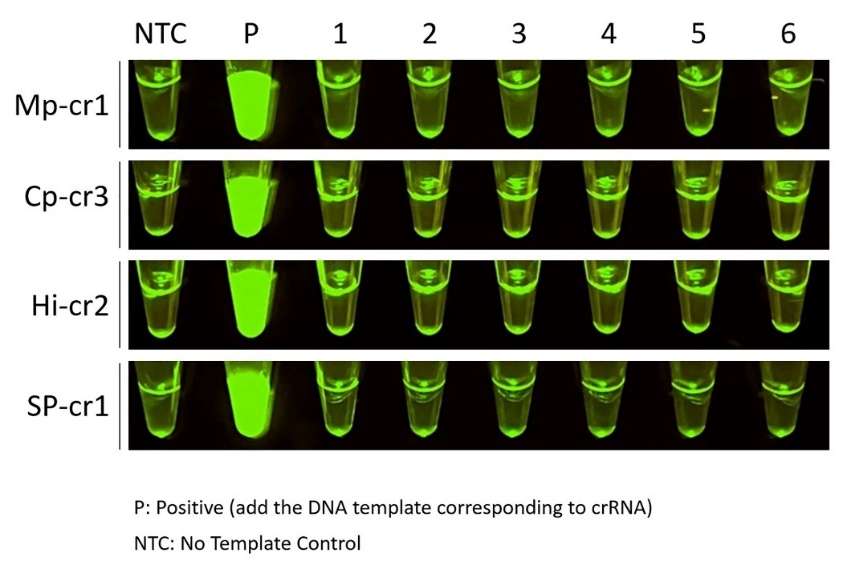


Supplementary Figure 1. Specific assay of the one-step RPA-CRISPR method in different bacterial strains. The one-step reaction system contains four preferred crRNAs that initiate the reaction when the DNA template binds to the crRNA. P, Positive (add the DNA template corresponding to crRNA); NTC, No Template Control; 1, *Klebsiella* *pneumoniae*; 2, *Pseudomonas* *aeruginosa*; 3, *Staphylococcus* *aureus*; 4, *Escherichia* *faecalis*; 5, *Escherichia* *coli*; 6 *Corynebacterium* *striatum.*


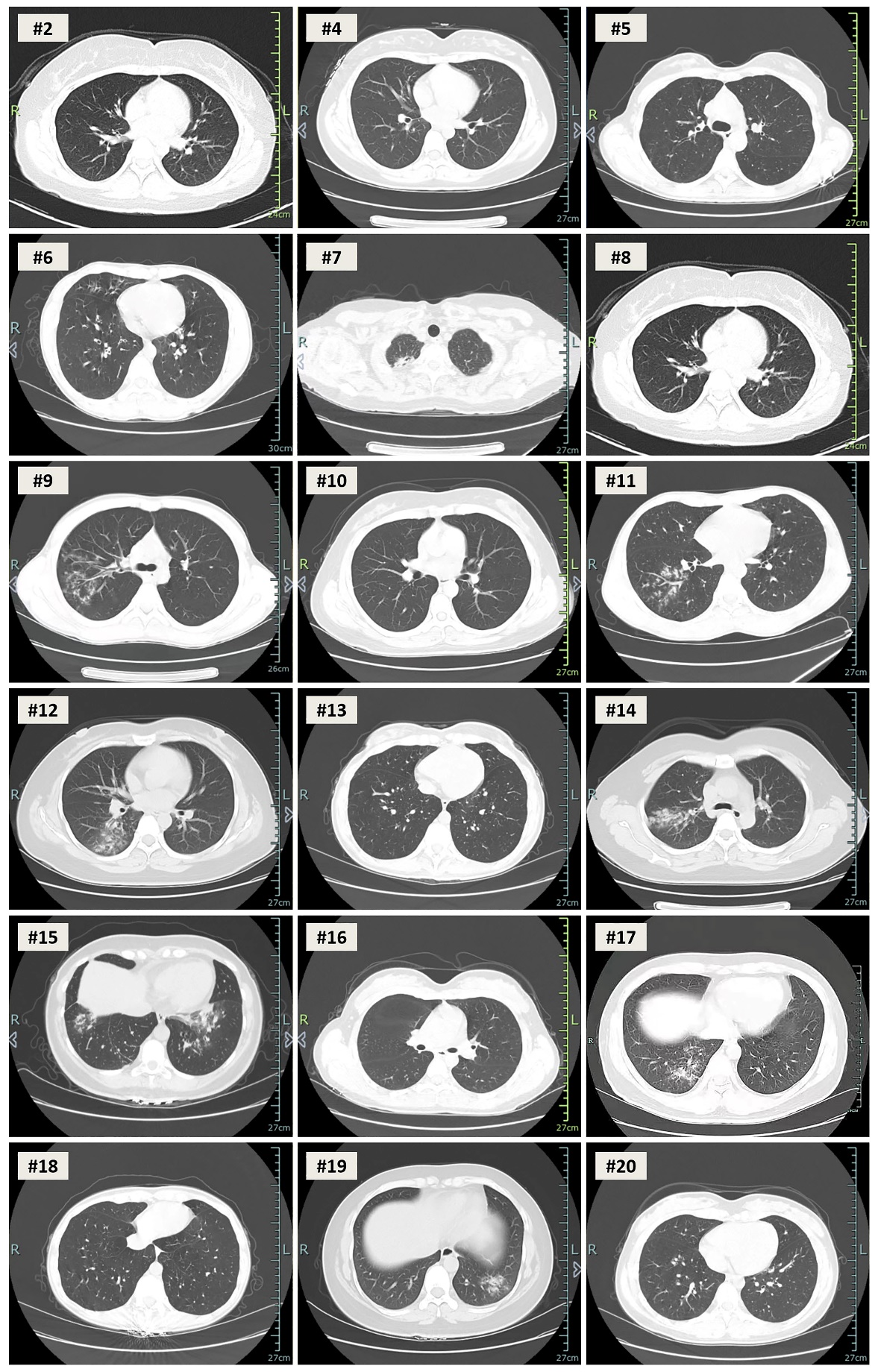


Supplementary Figure 2. Results of CT testing of throat swab samples. A total of 18 of the 20 throat swab samples are shown, and the remaining 2 samples representing healthy (#1) and infected (#3) are shown in Figure 7b.
